# Future Perspectives on Drug Targeting in Adult T Cell Leukemia-Lymphoma

**DOI:** 10.3389/fmicb.2018.00925

**Published:** 2018-05-09

**Authors:** Francesca Marino-Merlo, Antonio Mastino, Sandro Grelli, Olivier Hermine, Ali Bazarbachi, Beatrice Macchi

**Affiliations:** ^1^IRCCS Centro Neurolesi “Bonino-Pulejo”, Messina, Italy; ^2^Department of Chemical, Biological, Pharmaceutical and Environmental Sciences, University of Messina, Messina, Italy; ^3^Institute of Translational Pharmacology, The National Research Council, Rome, Italy; ^4^Department of Experimental Medicine and Surgery, University of Rome “Tor Vergata”, Rome, Italy; ^5^INSERM U1163, CNRS ERL 8654, Department of Hematology, Imagine Institute, Hôpital Necker-Enfants Malades, Paris, France; ^6^Department of Internal Medicine, American University of Beirut, Beirut, Lebanon; ^7^Department of Anatomy, Cell Biology and Physiological Sciences, American University of Beirut, Beirut, Lebanon; ^8^Department of System Medicine, University of Rome “Tor Vergata”, Rome, Italy

**Keywords:** HTLV-1, ATL, antiviral agents, biological therapy, targeted therapy

## Abstract

Human T cell leukemia virus type 1 (HTLV-1) is the etiological agent of adult T cell leukemia/lymphoma (ATL), HTLV-1 associated myelopathy (HAM/TSP), and of a number of inflammatory diseases with an estimated 10–20 million infected individuals worldwide. Despite a number of therapeutic approaches, a cure for ATL is still in its infancy. Conventional chemotherapy has short-term efficacy, particularly in the acute subtype. Allogeneic stem cell transplantation offers long-term disease control to around one third of transplanted patients, but few can reach to transplant. This prompted, over the past recent years, the conduction of a number of clinical trials using novel treatments. Meanwhile, new data have been accumulated on biological and molecular bases of HTLV-1 transforming and infecting activity. These data offer new rational for targeted therapies of ATL. Taking into account the double-face of ATL as an hematologic malignancy as well as a viral infectious disease, this Mini-Review seeks to provide an up-to-date overview of recent efforts in the understanding of the mechanisms involved in already used therapeutic regimens showing promising results, and in selecting novel drug targets for ATL.

## Introduction

**FIGURE 1:**
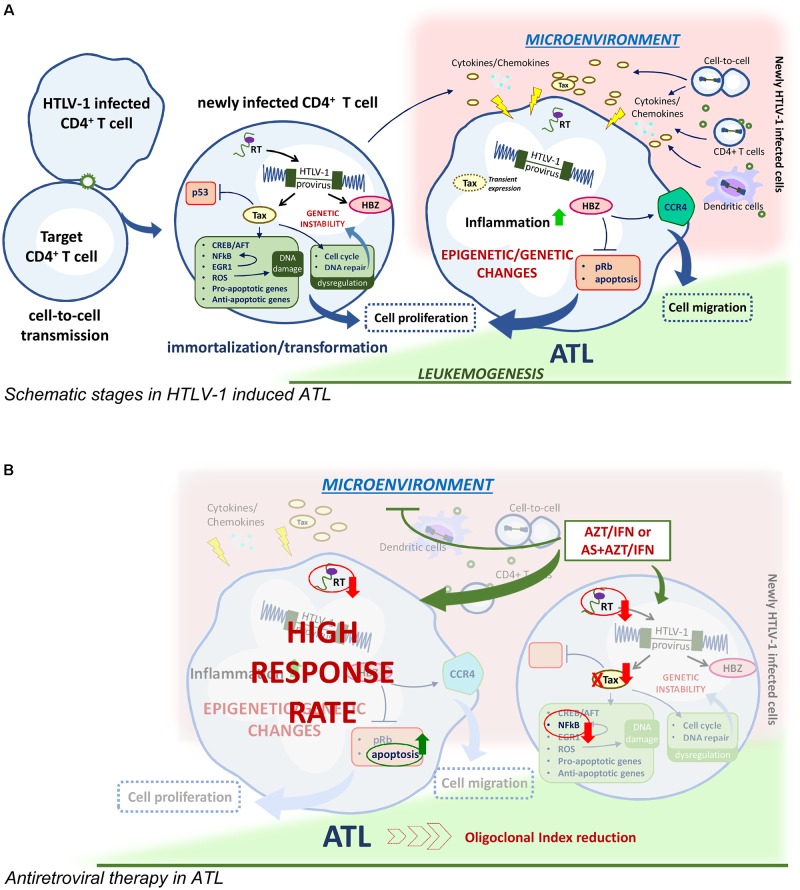
HTLV-1 driven leukomogenesis and possible mechanism of antiviral therapy. **(A)** Main immortalization/transformation process activated by HTLV-1 regulatory protein Tax and HBZ in newly infected CD4+ cells transmit virus to uninfected CD4+ cells and leading to epigenetic and genetic changes in ATL transformed cells. Cell migration and cell-to-cell virus transmission, presumably involving also dendritic cells, favor the release of inflammatory cytokines and chemokines milieu in the microenvironment and contribute to the maintaining of the infected clones. **(B)** AZT/IFN with or without arsenic trioxide (AS) could interrupt the maintaining route of ATL.

**FIGURE 2:**
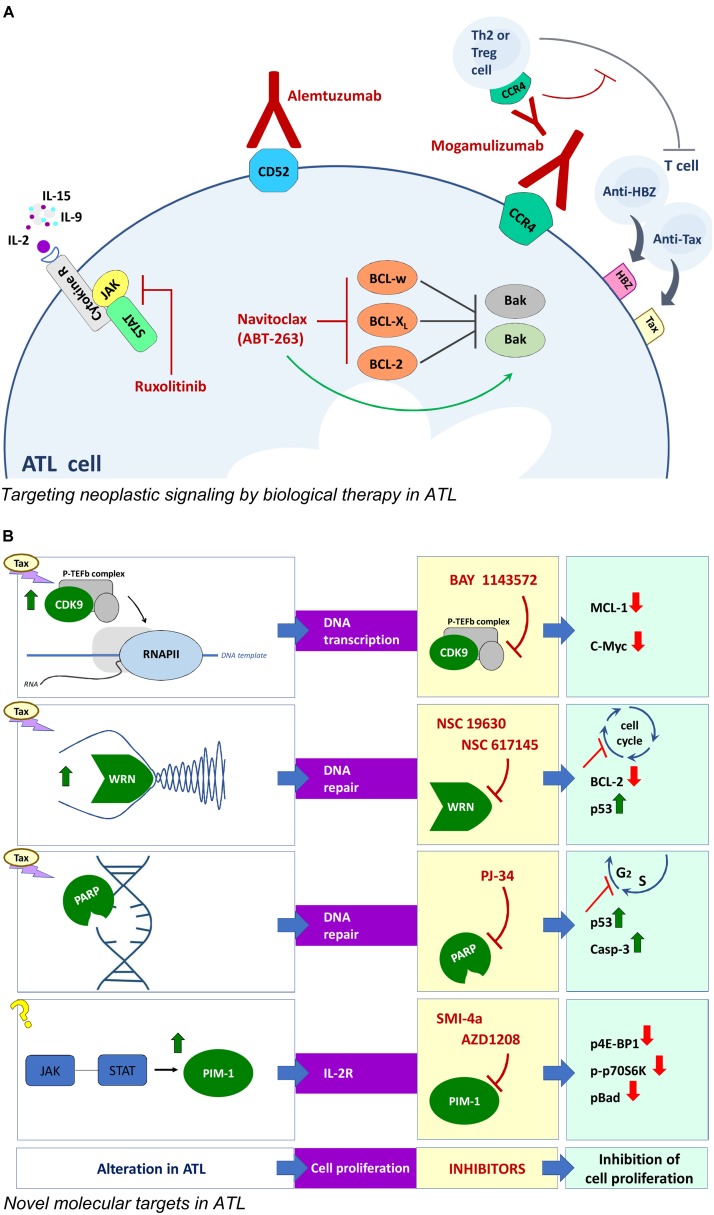
Novel approaches for ATL therapy. **(A)** Biological therapy with monoclonal antibodies and corresponding targets involved in neoplastic signaling in ATL cells. **(B)** Compounds and corresponding targets potentially useful for innovative targeted therapies in ATL.

Human T cell leukemia virus type 1 (HTLV-1) is the first identified human retrovirus endemic in southwestern Japan, the Caribbean islands, inter-tropical Africa, South America, Romania and the Middle East, with an estimated 10–20 million infected individuals worldwide. HTLV-1 is known to cause adult T cell leukemia/lymphoma (ATL), HTLV-1 associated myelopathy (HAM/TSP), and a number of inflammatory diseases. Firstly detected and isolated from a cutaneous T cell lymphoma almost 40 years ago, HTLV-1 still represents a significant challenge for the scientific community engaged to disclose its oncogenic potential and to identify a focused therapy (Gallo, 2005; Tagaya and Gallo, 2017). HTLV-1 transforms CD4+ lymphocytes *in vitro* and *in vivo*, and complex mechanisms control virus spreading, expression of viral proteins and host immune response in infected individuals (**Figure 1**). As a consequence, ATL patients are often refractory to intensive, conventional chemotherapy regimens. Classically used regimen are CHOP, CHOEP dose-adjusted EPOCH, and hyper-CVAD, alternating with high-dose methotrexate and cytarabine (Dittus and Sloan, 2017). An intensive treatment consisting of VCAP-AMP-VECP with the prophylactic use of G-CSF, has been introduced in Japan (Watanabe et al., 2011). In addition, results of preclinical studies, such as the high expression of CCR4 in ATL cells (Ishida et al., 2003), led to hypothesize new targets for biological therapy in ATL. Indeed, clinical trials using humanized monoclonal antibodies such as mogamulizumab (anti-CCR4) (Ishida et al., 2004; Yamamoto et al., 2010), alemtuzumab (anti-CD52) (Sharma et al., 2017), or daclizumab (anti-CD25) (Berkowitz et al., 2014) have been conducted on ATL patients (**Figure 2**). However, an important hurdle emerged in practically all the completed studies, is the limited duration of the response. Allogeneic stem transplantation could represent an alternative, potentially curative approach (Zell et al., 2016). Unfortunately, its use is limited to a small percentage of ATL patients. Recently published review articles provide detailed information on the state-of-the-art of treatment strategies until today adopted in clinical trials for ATL patients and on related results (Kato and Akashi, 2015; Hermine et al., 2018), and this mini-review will not address these aspects.

What makes the design of therapeutic approaches for ATL problematic is the double routes of HTLV-1 transmission *in vivo*, i.e., the mitotic and the RT-dependent ones, that might require a combined approach targeting both chronically infected host cells and direct viral replication. Actually, in the past recent years a lot of data have been accumulated on biological and molecular bases of HTLV-1 transforming as well as infecting activity. These data offer new rational for targeted therapies of ATL. Thus, taking into account the double-face of ATL as an hematologic malignancy as well as a viral infectious disease, this mini-review seeks to specifically focus on providing an up-to-date overview of recent efforts in: (i) understanding mechanisms involved in already used therapeutic regimens showing promising results, (ii) identifying and selecting novel drug targets for ATL.

## Oncogenic Potential of HTLV-1

Adult T cell leukemia/lymphoma is a severe, aggressive leukemia, unequivocally associated to HTLV-1 infection, which develop in 2–4% of the infected individuals after a long time of latency. Four clinical forms/subtypes of ATL have been recognized: acute, lymphomatous, chronic, and smoldering. (Shimoyama, 1991). The smoldering is the mildest form while the acute type represents the most aggressive form, but the life expectancy for each subtype is very poor reaching a maximum of 24 months. Although the molecular mechanism of virus transformation is highly complex and not entirely clarified, several studies highlighted that the viral regulatory proteins Tax and HTLV-1 bZIP factor (HBZ) play key roles in driving oncogenesis in HTLV-1 infected cells (**Figure 1**). The Tax protein has been demonstrated to trigger a number of immortalization/transformation related events in the early phase after infection, such as activation of the interaction with cAMP-responsive element-binding protein/activating transcription factor (CREB/ATF), the activation of NF-κB transcription factor, inhibition of p53, dysregulation of the cell cycle by interfering with the cellular checkpoint, impairment of cellular DNA repair mechanisms resulting in genetic instability, induction of DNA damage through production of reactive oxygen species, induction of both pro-apoptotic and anti-apoptotic activities (Chlichlia and Khazaie, 2010). In particular, Tax has been recently shown to intervene at an early phase of cell transformation by upregulating a family of early growth factor1 (EGR1), which upregulate the NF-κB system, establishing a positive feedback loop (Huang et al., 2017). Thus, Tax is likely required to initiate leukemogenesis while its expression is not routinely detected later in fresh cells from at least 50% of patients with established ATL. Recent data, however, demonstrated that survival of ATL cells depend on transient *tax* expression (Dassouki et al., 2015; Mahgoub et al., 2018) Conversely, HBZ is persistently expressed, even if at low level, *in vivo* in ATL cells, and interacts with elongation factors, Rb/E2F-1 complex, for cell cycle progression (Kawatsuki et al., 2016), inhibits apoptosis and upregulates expression of CCR4, thus promoting proliferation and migration of T cells (Sugata et al., 2016) and finally inducing global epigenetic changes in infected cells. In addition epigenetic dysregulation plays a role in ATL transformation consisting in GpC methylation of cell cycle, p53, apoptotic genes and histone modification of epigenetic reprogramming genes (Watanabe, 2017).

## Targeted Biological Therapy for ATL

Similarly to leukemic cells of different types, ATL cells exhibit high expression of genes associated to cell proliferation/death, cytokines, chemokines and/or markers of cell transformation. Therefore, shared potential pharmacological targets can justify in ATL the use of biological therapy set up for other malignancies. As observed in other neoplasia, the balance between pro and anti apoptotic response is subverted in ATL cells. Preclinical studies have shown that HTLV-1 infection *in vitro* gives rise, in a first phase, to high proliferation and a concomitant high apoptosis rate in infected cells until, in a successive phase, the selection of immortalized clones lead to outgrowth of cells preferentially exhibiting anti-apoptotic gene expression (Matteucci et al., 2004). Coherently, transformed clones from ATL patients over-express in culture the anti-apoptotic Bcl-2, Bcl-xL, and Bcl-w proteins and exhibit *ex vivo* a 10- to 20-fold higher sensitivity to navitoclax (ABT-263), an orally bio-available mimetic of the Bcl-2 homology domain 3 small molecule, as compared to non-HTLV-1-associated leukemic cells (Tse et al., 2008). Interestingly, molecular studies showed that the efficacy of navitoclax in ATL cells *in vitro* was increased by Tax induced upregulation of the pro-apoptotic Bax gene. However, the side effects of navitoclax limit its therapeutic use *in vivo*. Another crucial target in ATL can be detected in the complex network of autocrine (IL-2-IL-2Rα/IL-15-IL-15Rα) and paracrine (IL-9) loops, able to drive *ex vivo* spontaneous proliferation of ATL cells at an early stage (Chen et al., 2010). The three involved cytokines share in common a *γc* receptor whose expression is regulated by a family of kinase (JAK/STAT). Interestingly, JAK/STAT selective inhibitors suppressed the proliferation of smoldering/chronic ATL cells *ex vivo* (Ju et al., 2011). Given that combining inhibitors of the same signaling pathway can increase the chance to block cancer cell growth, a combination of navitoclax and of the JAK/STAT inhibitor ruxolitinib, was tested on ATL cells *ex vivo* and in animals. The combination provided additive/synergistic activity in inhibiting proliferation of ATL cells, delayed tumor growth and prolonged survival in tumor –bearing mice. This was associated to increasing inhibition of Bcl-xL which favored the upregulation of the pro apoptotic gene expression (Zhang et al., 2015). *In vivo*, the immune response has a remarkable impact on the turnover of HTLV-1 infected clones. Actually, cytotoxic T lymphocytes recognizing the immunodominant viral protein Tax and HBZ are both critical to determine the proviral load (Bangham and Matsuoka, 2017). Ruxolitinib is currently under evaluation in phase 2 clinical trials in ATL patients.

## Antiretroviral Therapy for Atl

Resistance to conventional chemotherapy prompted a reconsideration of therapy in ATL. Taking into account the retroviral etiology of the disease and that antiretroviral drugs proved their effectiveness in counteracting HIV infection, the use of AZT was tested in ATL. The rationale for this therapeutic intervention was to keep a low level of virus spreading and, possibly, also to limit the onset of inflammatory processes. In fact, successful results were initially reported in two preliminary phase II studies, using the combination of AZT and alpha interferon (IFN) showing an unexpected high response rate, particularly in previously untreated acute ATL patients (Gill et al., 1995; Hermine et al., 1995, 1998). A prospective phase II study on 13 patients who received AZT/IFN treatment as initial therapy, showed nine complete response (CR) and four partial remission with mild toxicity. The CR patients survived more than three years, after which most of the patients relapse underlying the need for additional therapy with AZT/IFN (Hermine et al., 2002). The impact of first-line AZT/IFN therapy on long-term survival was reported in a worldwide meta-analysis showing a significant improvement of survival of the leukemic subtypes with an unprecedented 100% 5-year overall-survival in chronic and smoldering ATL, at a median follow-up time of 5 years (Bazarbachi et al., 2010). Although AZT/IFN therapy provided reasonable management of ATL, most patients relapse. To counteract this aspect, based on previous experience in acute promyelocytic leukemia, arsenic trioxide (AS) was tested in ATL. The arsenic/IFN combination induced proteasomal degradation of Tax through stepwise poly-sumoylation and SUMO-dependent ubiquitination (El-Sabban et al., 2000; Dassouki et al., 2015). This combination cured murine ATL derived from tax-transgenic through selective targeting of ATL leukemia initiating cells (El Hajj et al., 2010). The triple combination of arsenic, IFN and AZT resulted in a high rate of response in chronic ATL patients (Kchour et al., 2009). However, the mechanisms involved in the therapeutic effectiveness of AZT/IFN are not clear, although *in vitro* studies demonstrated that this combination differently affects HTLV-1 mRNA and viral protein expression and activates the p53 pathway and apoptosis in HTLV-1 infected cells (Kinpara et al., 2013). Nevertheless, no clear evidence was provided concerning the inhibition of viremia by AZT/IFN in ATL patients. However, viremia in ATL patients is low and viral load, reverse transcriptase (RT) activity and/or other virus related assays, carried out in lymphocytes from patients, could be more reliable parameters for assessing HTLV-1 replicative potential in ATL patients. Interestingly, we have recently reported that long-term *in vivo* therapy with AZT and IFN actually caused complete inhibition of RT activity, reduction of p19 release and viral mRNA, and a dramatic decrease of the oligoclonal index, in short-term cultures of PBMCs from ATL patients who responded to therapy, but not in those who did not respond (Macchi et al., 2017). Thus, the above reported data sustain that the therapeutic efficacy of AZT/IFN combination in ATL is actually mediated, at least in part, by the inhibition of RT-dependent viral replication. Consequently, we can hypothesize that the AZT/IFN combination in ATL patients targets viral replication presumably outside leukemic cells. This could occur in cells such as dendritic cells or in newly infected T lymphocytes, immediately after their first contact with the virus, or other cell types in which a dynamic, continuous viral replication occurs. In this case, AZT/IFN treatment can impede HTLV-1 viral replication that could provide a microenvironment that is mandatory for survival and/or renewal of ATL cells (**Figure 1**). This could occur through direct cell-to-cell communication or paracrine stimulation through secreted Tax or various cytokines/chemokines as reported for chronic lymphocytic leukemia (Bazarbachi et al., 2011). Moreover, these findings could explain the impossibility to set-up long-term culture of ATL cells *in vitro* and why the AZT/IFN combination exerts a beneficial effect *in vivo* but not *ex vivo* on ATL cells. A recent trial using EPOCH chemotherapy in combination with bortezomib, to block NF-κB activation, and raltegravir, as antiviral drug, in acute ATL and in ATL lymphoma showed that this regiment was well tolerated, leading to 67% response rate. Changes in RNA viral load and HBZ viral expression *ex vivo* were found as reliable parameters of response as well as inhibition of viral replication and repression of NF-κB activation through proteasome inhibition (Ratner et al., 2016). Thus, accumulating evidence sustains that controlling virus spread is a crucial aspect in ATL therapy.

Regarding *in vitro* studies, AZT and tenofovir inhibit virus transmission to PBMC in short-term cultures and interfere with immortalization in the long run at a drug concentration which was poorly toxic toward uninfected cells (Macchi et al., 1997; Balestrieri et al., 2005). Conversely, lamivudine was not effective in inhibiting HTLV-1 infection *in vitro*, presumably owed to the presence of an isoleucine at position 118 in HTLV-1 RT, conferring natural resistance to 3TC (Balestrieri et al., 2002; Toro et al., 2003). Different not licensed compounds, such as the PCOAN phosphonates, were able to inhibit cell-to-cell HTLV-1 transmission directly inhibiting the HTLV-1 RT activity, as demonstrated by a cell-free assay (Balestrieri et al., 2008b). Inhibition of HTLV-1 infection could also rely on other still unclear mechanisms, as shown in case of compounds of natural origin such as carbohydrate-binding agents (Balestrieri et al., 2008a) and an extract from the seeds of bergamot, which remarkably blocked virus horizontal transmission *in vitro* (Balestrieri et al., 2011). In addition, raltegravir and diketo acid, MK-2048, were active inhibitors of viral transmission as well as viral immortalization of HTLV-1 in lymphoid and non lymphoid cells, *in vitro* (Seegulam and Ratner, 2011).

## Novel Molecular Targets in Atl

Further targets are being investigated to find new therapeutic approaches in ATL (**Figure 2**). Tax is known to remarkably affect the host cell proliferation by directly intervening in the processes regulating DNA transcription, replication and repair. On the basis of this regulatory role of Tax in HTLV-1 transformation, a few druggable targets have been demonstrated in preclinical studies. CDK9 is a component of a transcription factor, P-TEFb, essential for transcription of most MHC class II genes and for the transcriptional elongation by phosphorylating the C-terminal domain of RNA polymerase II. The importance of CDK9 for a targeted therapy was demonstrated in advanced stage of chronic lymphocytic leukemia and multiple myeloma (Tong et al., 2010). An additional reason to investigate CDK9 as a possible molecular target in ATL is that P-TEFb is present within HTLV-1 transformed cells in Tax-regulated complexes (Cho et al., 2010). In fact, the P-TEFb/CDK-9 inhibitor, BAY 1143572, was able to block the growth of ATL cells *ex vivo*, and to decrease MCL-1 and c-Myc expression levels. In addition, BAY 1143572 decreased ATL cell migration in the liver and bone marrow in a model of ATL *in vivo* xenograft, in immunocompromised NOG mice (Narita et al., 2017). Tax was reported to affect the replicative fork during DNA replication by blocking the progression of the process (Chaib-Mezrag et al., 2014). Helicases are deeply involved in DNA double-strand break repair through the homologous repair as well as the non-homologous end-joining pathway. In particular, the WRN helicases are mutated in cancer and are generally highly expressed in human leukemia (Sallmyr et al., 2008). This finding prompted to assay WRN helicases inhibitors in ATL cells. The results showed that the WRN inhibitors NSC 19630 and NSC 617145 efficiently killed HTLV-1-transformed and patient-derived cells, by inducing cell cycle arrest, downregulation of BCl-2, caspase 3 activation, and apoptosis in a dose-dependent manner, without affecting HTLV-1 expression (Moles et al., 2016). Tax was also recognized to induce genomic double strand breaks during DNA replication and alteration in the subsequent use of non-homologous end joining pathway for repair during the S phase (Baydoun et al., 2012). Thus, the genomic instability afforded by Tax represents a possible target within the repair enzymes family. PJ-34, a small molecule inhibitor of poly (ADP-ribose) polymerase (PARP), arrested cell cycle at S/G2M phase, inducing reactivation of p53 and caspase 3 activation in HTLV-1 infected cells. However, MT-2 chronically infected cells were resistant to PJ-34, showing reduced caspase 3 cleavage and increased RelA/p65 expression. These results suggest that the NF-κB system might be involved in resistance to PJ-34 (Bai et al., 2015). The inhibitors of JAK/STAT pathway regulating expression of IL-2R common γ chain reported as therapeutic option in ATL, were found to be highly immunosuppressive. Thus more recent data proposed a different JAK/STAT-pathway associated target, relying on the Pim 1 downstream target of JAK, whose expression is regulated by miRNA124a. The Pim 1 gene was found to be constitutively expressed in 71% of freshly isolated ATL cells and in chronically HTLV-1 infected cell lines, while PBMC from healthy donors were negative. Treatment with the Pim 1 inhibitors, SMI-4a or AZD1208, decreased ATL cells proliferation and decreased Pim 1 activity as demonstrated by downregulation of p4E-BP1, p-p70S6K, and p-Bad. The AZD 1208 was found more efficacious than SMI-4a. Moreover, AZD 1208 significantly inhibited ATL tumors in the pre-clinical NOG mice model (Bellon et al., 2016), showing that the JAK/STAT-Pim1 pathway could be a novel therapeutic target for the treatment of ATL. In addition the ATL cells exhibited a downregulation of miRNA and Dicer expression. The suitability of these target was demonstrated by the fact that the *in vitro* effect of deacetylase inhibitor, valproate, on ATL cells was owed to the rescue of the pre-miRNA maturation pathway (Gazon et al., 2016).

## Conclusion

Theoretically, ATL cells exhibit a number of potential, different viral and cellular pharmacological targets. A number of studies suggests to take under consideration the suitability of numerous, known drugs to counteract ATL. However, it is hard to explain why, despite broad chances of potentially druggable targets, success is still limited in ATL clinical studies. Possible reasons could reside in the long latency of HTLV-1 infection, allowing the virus to escape host response, as well as the lack of suitable markers of disease progression. Hopefully, more deep knowledge of how the virus affects the regulation of host immune response and the metabolic requirements of transformed cells could represent new issues for future challenges in ATL therapy.

## Author Contributions

FM-M searched for literature, wrote the paper, and performed the elaboration and graphical representation of the figures. AM, AB, and BM searched for literature and wrote the paper. SG searched for literature. OH revised the paper.

## Conflict of Interest Statement

The authors declare that the research was conducted in the absence of any commercial or financial relationships that could be construed as a potential conflict of interest.
